# Application of Metal-Based Catalysts for Semi-Hydrogenation of Alkynol: A Review

**DOI:** 10.3390/ma16237409

**Published:** 2023-11-28

**Authors:** Pengxian Wang, Yue Ma, Yiran Shi, Fangying Duan, Meng Wang

**Affiliations:** 1 Queen Mary University of London Engineering School, Northwestern Polytechnical University, Xi’an 710129, China; 2 State Key Laboratory of Solidification Processing, School of Materials Science and Engineering, Northwestern Polytechnical University, Xi’an 710072, China; 3 School of Materials Engineering, Xi’an Aeronautical University, Xi’an 710077, China

**Keywords:** semi-hydrogenation, alkynol, metal catalysts

## Abstract

Alkynol semi-hydrogenation plays a vital role in industrial processes, due to the significance of its main product, enol, in high-end chemical synthesis, such as medicine, pesticide, food additives, and polymer monomer synthesis. Multiple intermediates are formed through a complex series of parallel or continuous reactions under varying conditions. However, the selectivity and efficiency of catalysts for producing these products still pose significant challenges. This review aims to thoroughly discuss the challenges and advancements in catalysts using different species and supports under various reaction conditions. Furthermore, strategies to enhance the yield and rate of enols are summarized based on noble metals, non-noble metals, and metal comparisons. By addressing diverse catalysts and reaction conditions, this review provides valuable insights into improving the semi-hydrogenation of acetylenic alcohols to enols.

## 1. Introduction

Enols produced through the selective semi-hydrogenation of acetylenic alcohols exhibit unique chemical properties and are significant materials in the synthesis of pharmaceuticals, agricultural products, fragrances, and flavors in industrial processes [1,2]. For instance, cis-2-butene-1,4-diol (cis-BED) serves as a key intermediate in various synthetic pathways and is utilized in the production of endosulfan, vitamin A, and vitamin B6. Additionally, it acts as a monomer in the formation of poly (2-butene diol), an unsaturated telechelic polyether diol with remarkable potential. Similarly, 2-methyl-3-buten-2-ol (MBE), a vital compound in the synthesis of vitamin A, vitamin E, and luxury fragrances, is essential [3]. Finally, cis-3-hexen-1-ol represents an important intermediate with a strong grassy aroma, widely used in the production of common chemicals and food flavors [4].

As suggested by its name, the semi-hydrogenation of acetylenic alcohols is a catalyst-mediated reaction that involves converting carbon–carbon triple bonds into valuable enol products. This process is continuous and comprises several key steps. First, the catalyst is synthesized and adsorbed onto a support. Next, it reacts with H_2_O, H_2_, or alcohols in order to adsorb H atoms on its surface. Subsequently, with the supply of external energy (usually achieved through heating, pressurizing, providing electric current, or using light energy), the carbon–carbon triple bond is partially broken and undergoes an addition reaction with H on the catalyst, forming an intermediate. Finally, the intermediate combines with H on the catalyst to generate the final product, the enol, while the catalyst is re-reduced and enters the cycle [5,6,7].

The addition of carbon–carbon triple bonds to H is a critical step in achieving accurate semi-hydrogenation of acetylenic alcohols and producing valuable enols. This process is mainly influenced by thermodynamic and mechanistic factors [8,9]. If the addition of adsorbed hydrogen favors enols over alkanols, meaning that the equilibrium constant for enol formation is significantly greater than that for alkanol formation, incentive selectivity dominates. On the other hand, thermodynamic selectivity is at play when the catalyst preferentially adsorbs acetylenic alcohols in the presence of alkenes, thereby occupying active sites without excessive hydrogenation to form alkanols [10].

In this article, catalysts are broadly categorized into two groups for discussion: noble metal catalysts and non-noble metal catalysts. Among noble the metal catalysts, palladium-based catalysts like the Lindlar catalyst set a high standard, due to their high catalytic rates and selectivities [11]. However, the use of gaseous hydrogen resources and toxic Pn significantly limits the development of selective hydrogenation [7]. Conversely, non-noble metal catalysts present a different set of advantages and challenges, which warrant exploration. This review aims to provide a comprehensive perspective on these two classes of metal catalysts, examining their mechanisms, reaction conditions, advantages, and potential challenges. As the field of catalysis continues to progress, we will also delve into emerging catalysts, heralding a new era of semi-hydrogenation. Our objective is to offer a comparative analysis of noble metal catalysts and non-noble metal catalysts, serving as a valuable reference for further advancements in the research and application of acetylenic alcohol semi-hydrogenation.

## 2. Semi-Hydrogenation of Alkynols over Noble Metal Catalysts

### 2.1. Pd-Based Catalysts

Pd-based catalysts are widely used in the industrial production of acetylenic alcohols. In the following section, we will provide examples of the mechanisms and evaluate the strengths and weaknesses of these catalysts, in terms of their thermal, electrocatalytic, and photocatalytic properties. A comparative analysis will be presented to assess the characteristics of these catalysts.

#### 2.1.1. Lindlar Catalyst (Pd-Pb/CaCO_3_)

**Figure 1:**
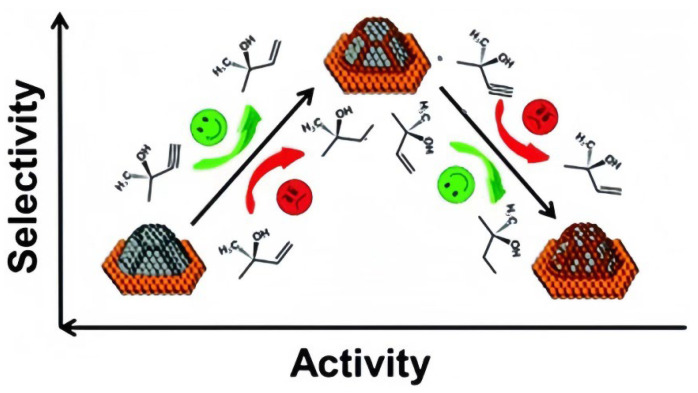
Lindlar selective poisoning mechanism [1]. Copyright 2019, Royal Society of Chemistry.

The Lindlar catalyst is a Pd-based catalyst renowned for its high catalytic rate and selectivity (Figure 1). This selective catalytic hydrogenation catalyst is composed of approximately 1.8 wt% of Pd [12]. The principle behind the Lindlar catalyst is relatively straightforward. Alkynes exhibit greater reactivity than alkenes under normal conditions, enabling them to stably adsorb onto the catalyst’s surface. However, once they convert to enols, their adhesion to the catalyst decreases, leading to deactivation. To prevent further conversion of alkenes to alkanes, heavy metals are employed to poison the catalyst, effectively compartmentalizing reactants and products. This approach achieves alkynol conversion rates of up to 99% [13]. The following equations can be utilized to calculate enol selectivity, yield, and conversion, where M represented mass:

$$S_{Alkenol}=\frac{Yield}{Conversion}\;Yield=\frac{M_{Actual\;alkenol}}{M_{Theoretical\;alkenol}}\;Conversion=\frac{M_{ALKENOL}}{M_{ALKYNE}}$$

By selectively poisoning the active site, the selectivity can be further heightened to 98–99% [1]. This method represents an extraordinarily efficient form of catalysis. The reaction takes place in a conventional thermocatalytic process, typically utilizing a Parr autoclave under reaction conditions of 80 degrees Celsius and 0.1 MPa. However, the drawbacks associated with Lindlar catalysts are noteworthy. Poison-induced enol is challenging to employ in medical and biological applications, significantly limiting the utility of the Lindlar catalyst.

#### 2.1.2. Pd-B Catalyst

**Figure 2:**
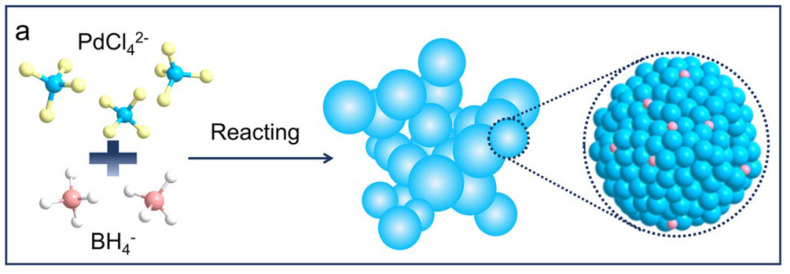

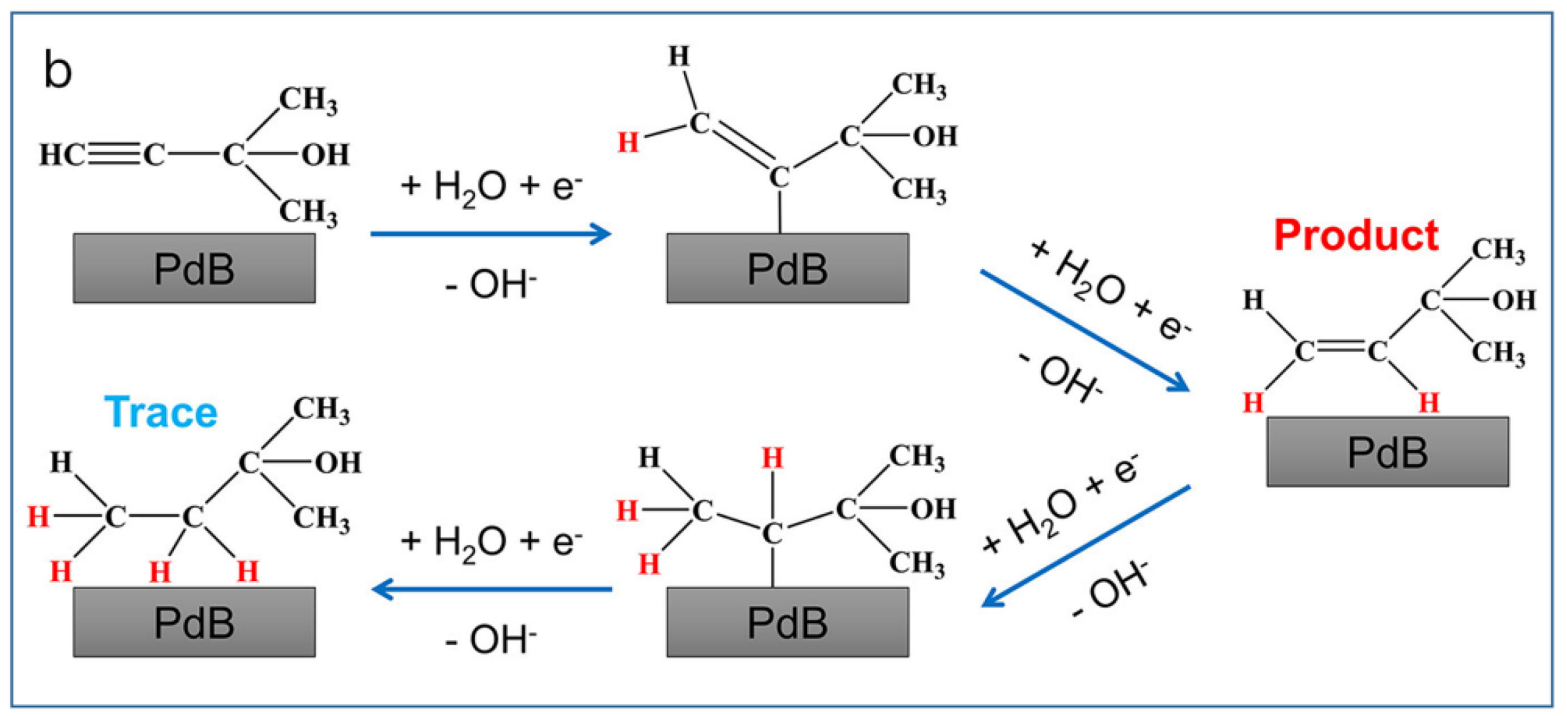
(**a**) Composition of the Pd-B catalyst; (**b**) the reaction process of ethynol [7]. Copyright 2023, American Chemistry Society.

Pd-B catalyzed semi-hydrogenation has emerged as a novel approach for synthesizing alkenols, offering advantages over the traditional Lindlar catalytic method. This catalytic approach ensures high yields and selectivity, without the drawbacks of heavy metal poisoning. Two notable features distinguish this method. First, it utilizes electrocatalysis instead of conventional thermal catalysis, resulting in lower energy requirements [14]. Second, unlike H_2_, Pd-B electrocatalysis employs hydrogen sourced from water. Examining the diagram below (Figure 2a,b) reveals that Pd-B catalyst particles exhibit numerous branches, increasing the contact area between the catalyst and reactants and thereby enhancing the reaction efficiency. The concentration of Pd-B in the catalyst is approximately 0.6 wt%. During the reaction, one carbon atom from the triple bond of the alkyne alcohol first attaches to the catalyst. Through energy input, water combines with an electron to generate a hydroxide ion and a hydrogen atom. In order to achieve bond stability, the other carbon atom on the original triple bond incorporates this hydrogen atom. Consequently, an enol compound is produced, with a potential of −1 V, yielding a theoretical conversion of 90.2% and a selectivity of 94.5 [7]. The foremost advantage of Pd-B electrocatalytic urea synthesis lies in its ability to circumvent the high pollution and energy consumption associated with thermal catalysis. Additionally, the resulting products possess a wider range of applications.

#### 2.1.3. Pd-C Catalyst

**Figure 3:**
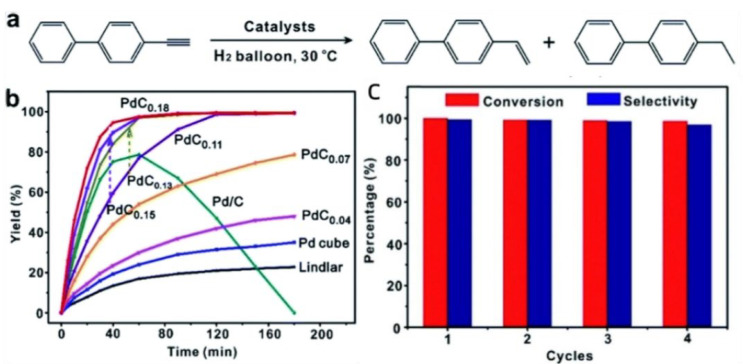
(**a**) Catalytic reaction equations for Pd-C catalysts; (**b**) time to complete catalysis for different catalysts; (**c**) catalytic stability and selectivity of PdC0.18 catalyst [15]. Copyright 2019, Royal Society of Chemistry.

Pd-C catalysts exhibit higher selectivity compared to other Pd-based catalysts in the semi-hydrogenation reaction of alkyne alcohols (Figure 3a). Ensuring the stability of Pd-C during catalysis presents a significant challenge. Guo et al. conducted experiments utilizing glucose treatment of Pd crystals to achieve stability under continuous catalysis. The results demonstrated a turnover frequency of 7896 h^−1^, while maintaining over 99% selectivity, indicating the capability of conducting the reaction on a large scale within a short timeframe. Pd-C catalyst, belonging to the Pd-based catalyst family, displays superior selectivity compared to the other variants (Figure 3b) [15]. The proportion of carbon (C) in the crystals significantly influences the catalytic efficiency of Pd-C catalyst, with an optimal molar ratio of 1:0.18 based on experimental findings (Figure 3c). Nikoshvili et al. additionally found that employing mesoporous Pd catalysts was advantageous for catalyzing long-chain alkynols [16].

#### 2.1.4. PdZn/Meso_S-C Catalyst

**Figure 4:**
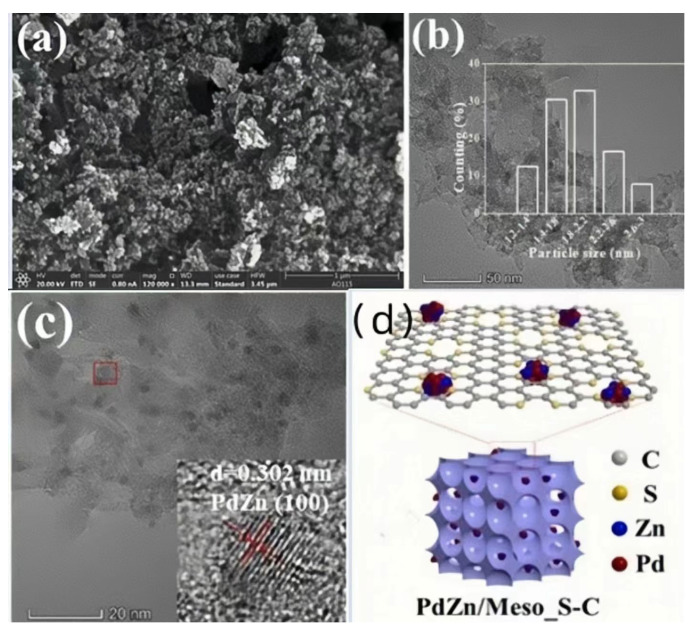
(**a**) SEM image of Meso_S-C; (**b**) low-magnification TEM image of PdZn/Meso_S-C and histogram of the distribution of PdZn NPs; (**c**) HR-TEM image of PdZn/Meso_S-C; (**d**) structure diagram of the PdZn/Meso_S-C catalyst [16]. Copyright 2022, MDPI.

The addition of elemental sulfur to PdZn/Meso_S-C disperses the PdZn bimetallic particles (Figure 4a–d), increases the rate and stability of the reaction, and slightly improves the selectivity and yield compared to conventional bimetallic catalysts. In the experiments of Hanghang Zhu’s team **[16]**, we can see that the catalyst increased the conversion of the reactants to 97.5% and had 96% selectivity for propargyl alcohol ethoxylates (Table 1). Highly efficient catalysis was achieved. Incidentally, they also gave case data on the reactions during the experiment. This method also has its limitations. For example, the catalyst preparation process is energy-intensive and cumbersome. The PdZn catalyst used is also costly.

#### 2.1.5. Pd-Enhanced Alkynol Hydrogenation on msAC

In a pioneering study by Oberhauser et al., a carbon-rich feedstock was utilized to craft an activated carbon (msAC) via a straightforward carbonation technique. This served as an adept support for palladium (Pd) nanoparticles in acetylene hydrogenation. Impressively, the 0.25% Pd/msAC catalyst showcased a near-perfect acetylene conversion rate of ~99.9% and ethylene selectivity of ~99.9%, while demonstrating resilience over 100 h at 260 °C. The distinctive pore architecture of msAC ensured the optimal dispersion of Pd nanoparticles and emphasized the Pd (111) crystal plane’s exposure, highlighting the invaluable potential of M. sinensis biomass in devising superior catalysts [17].

#### 2.1.6. Other Catalysts

Pd/Al_2_O_3_ catalyst represents another approach for the semi-hydrogenation of alkynols. Alberto and team explored the reaction conditions and yields associated with this method [18]. Unlike typical liquid-phase hydrogenation, this reaction occurs in the gas phase, facilitating the extraction of target products. The catalyst demonstrates exceptional performance in the catalysis of four-carbon alkynols. Iain T. Duncanson and team utilized Pd-C catalysts to enhance the original scheme, resulting in increased conversion rates in the liquid phase [18]. Although this is a conventional thermal reaction, it requires highly pure reactants. Additionally, Pt-Pb bimetallic electrocatalysis has proven to be an effective method for semi-hydrogenation, particularly in the production of Z-enols. This process employs a PDM reactor, which facilitates efficient and orderly reactions. However, there are certain drawbacks associated with this method, such as the use of heavy metal additives in the manufacturing process and the formation of Z-E isomers [19].

Bu and team employed Cu nano-monoliths to achieve monometallic electrocatalysis, resulting in high yields of 93%, while enabling mass production [20]. In a complementary approach, they synthesized polymer-supported PdCu alloy nanoparticles with a 1:1 atomic ratio of palladium to copper, via coordination of metal acetates to 2,2′-bipyridine-functionalized poly (lactic acid) (PLA), followed by stereocomplexation of PLA-based macrocomplexes and metal reduction with hydrogen. These nanoparticles exhibited high efficacy in the semi-hydrogenation of key industrial alkynols, achieving up to 98% selectivity for the corresponding cis-alkenol under mild conditions without any additives. Furthermore, when compared to isolated Pd and Cu in the same chemical environment, the supported PdCu nanoparticles demonstrated a pronounced alloy effect, ensuring high chemoselectivity for the alkene at elevated alkyne conversions. Recycling tests reaffirmed the catalyst’s robustness and stability, even upon exposure to air. This offers a promising and efficient catalytic solution for industrial applications [21].

Photoanode position-assisted semi-hydrogenation is another catalytic approach, with Pd@Ag/TiO_2_ particle catalysts serving as the central focus of the experiments. Light is provided by a high-pressure mercury lamp [22]. The investigation proposed by Maazaoui et al., on the other hand, changed the original catalyst of the market, synthesizing a novel Cl_2_Pd(PPh_3_)_2_ catalyst, which originated from the interaction between Zn^0^ and ZnI_2_, while maintained in an atmosphere of H_2_, achieving a high catalytic efficiency under milder conditions [23]. Finally, the specific properties of each catalysis method are listed in Table 2.

### 2.2. Pt-Based Catalysts

Pt and Pd exhibit similar properties, despite some notable differences. For instance, Pt demonstrates a lower hydrogen affinity compared to Pd and is less stable at elevated temperatures. These characteristics impose limitations on the utilization of Pt in thermal catalysis. However, Pt’s advantageous attributes, such as excellent electrical conductivity and stable pricing, have established it as a catalyst of choice for alkynol catalysis. The following section presents three specific examples.

#### 2.2.1. Pt{hkl} Catalysts

**Figure 5:**
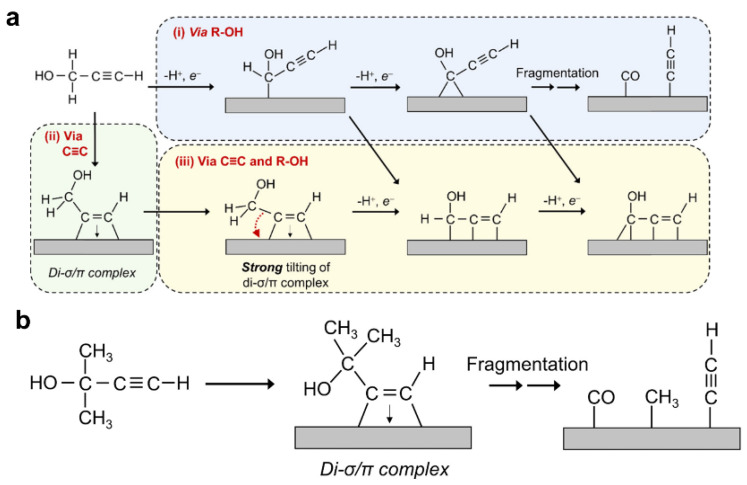
(**a**) Reaction mechanism of PA on Pt catalyst surface [15,16]; (**b**) reaction mechanism of MeByOH on Pt catalyst surface [33]. Copyright 2000, Elsevier.

Pt{hkl} single crystal electrodes are prepared, and Pt is subsequently used to adsorb hydrogen, resulting in a sequential reaction. The extent of this reaction (Figure 5) can be evaluated through current density and spectral analysis. Both diagrams exhibit a notable characteristic where the carbon atom on the main chain forms either a σ or π bond with the Pt metal, initiating further reactions. The ultimate product is generated in Reaction III. Experimental findings from Guan’s group indicated that Pt{100} displayed a higher activity than Pt{110} within the potential range of 0 to 0.8 V, while Pt{111} exhibited the lowest activity [32]. The relatively low activation of Pt{111} serves to prevent excessive activation, which may lead to the production of substantial by-products, such as CO. Consequently, employing Pt{111} offers the most favorable approach to achieving enhanced selectivity and higher yields.

#### 2.2.2. PtCl_2_/XPhos Catalyst System

PtCl_2_/XPhos catalyst is prepared by combining a specific quantity of PtCl_2_ with XPhos. For instance, in the synthesis of but-3-yn-2-ol, a 5 mol% PtCl_2_ to 10 mol% XPhos ratio is utilized. The PtCl_2_/XPhos catalysts enable the reaction of alkynyl alcohols with silanes, resulting in the formation of alkenols with silyl groups [34]. Subsequently, the silyl groups are substituted with hydrogen, to yield the final enol.

The reaction equation demonstrates the production of the Z-E isomer of alkynyl alcohol after the process of hydrogenation, wherein the β isomer is the primary product. This main product can be selectively isolated using spectral analysis and desilylated to obtain the enol product. Dario Braga’s investigation [35] revealed that alkynyl alcohol forms a hydrogen bond with Pt, which is significantly long-reaching up to 4.2 nm. Moreover, the bond angles align with the conditions required for semi-hydrogenation, favorably facilitating the reaction.

#### 2.2.3. Pt/SiC Catalyst

Pt/SiC catalyst is a thermocatalytic catalyst that utilizes SiC as the primary carrier, incorporating Pt. It functions similarly to the Pd/SiC catalyst but offers additional advantages. For instance, when aiming for higher reaction rates, the proportion of Pd in the catalyst must be increased in the case of Pd/SiC. In fact, for industrial production with Pd/SiC, a Pd content ranging from 2 to 5 wt% is often necessary. In contrast, experiments conducted by Shu et al. [36] demonstrated that high reaction rates can be achieved with Pt catalysts containing only 0.5 wt% Pd.

**Figure 6:**
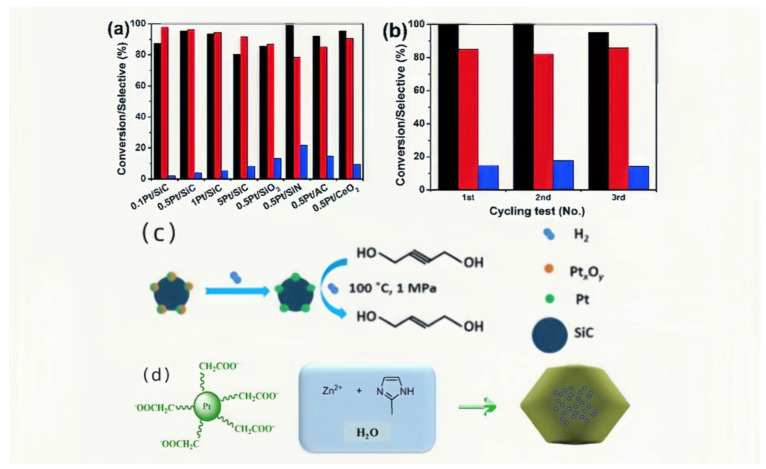
(**a**) Selectivity and conversion of different catalysts [37]; (**b**) recyclability of 0.5Pt/SiC at 100 °C and p(H_2_) = 1 MPa (black: conversion of BYD; red: selectivity of BED; blue: selectivity of BDO) [36]; (**c**) Pt/SiC conversion as catalyst in reactions [37]; (**d**) manufacturing process of Pt@ZIF-8 [38]. Copyright 2016, Elsevier.

Figure 6a–d depicts the reaction mechanism, wherein oxidized platinum forms platinum and stores hydrogen by combining with hydrogen on SiC. Subsequently, it is re-oxidized, while reacting with a carbon–carbon triple bond, resulting in the reduction to a carbon–carbon double bond and entering the cycle.

Shu et al. also conducted experiments on catalysts with varying temperatures and Pt contents, identifying the group that exhibited the highest reaction yields and selectivity within a 10 h span. The Pt content in this group was 0.5 wt%. The experimental data for this specific group are presented below. The reactant used in their experiments was 2-butyne-1,4-diol, leading to a 96% conversion of Alkynol and a 96% selectivity of Enol catalyzed by Pt/SiC [37]. Furthermore, the experiments conducted by Li et al. revealed that, by employing Pt@ZIF-8 as the catalyst, a remarkable 99% conversion of Alkynol and a 92% selectivity of Enol were achieved [38].

#### 2.2.4. Platinum NPs by *Eschericha coli*

**Figure 7:**
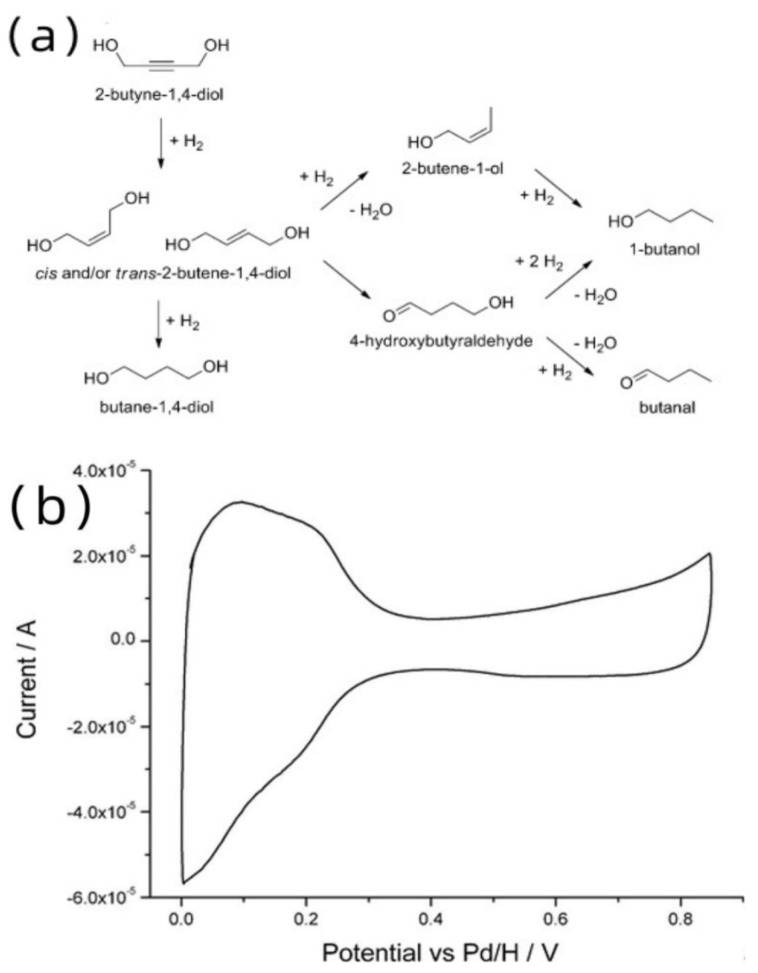
(**a**) Reaction process of 2-Butyne-1,4-diol. Copyright 2012, American Chemistry Society. (**b**) CV recorded for precleaned 20% bio-Pt (derived from *E. coli*) supported on a glassy carbon electrode, in 0.1 M sulfuric acid, sweep rate 50 mV/s [36]. Copyright 2012, American Chemistry Society.

Bennett’s group [36] proposed a novel approach, instead of solely focusing on enhancing the chemical structure and distribution state of the catalyst. Figure 7a presents the conversion processes of 2-Butyne-1,4-diol into a range of products, including the major 2-Butene-1,4-diol from the point of view of the reaction mechanism. Figure 7b reflects the rate of this reaction from the point of view of the electric current. They utilized the Pt catalyst as a feedstock to induce toxicity in E. coli, subsequently isolating the catalyst from the cytoplasm for further usage. The resulting Pt catalysts demonstrated a higher activity compared to industrially produced catalysts, leading to increased reaction rates. This method can potentially serve as an alternative to the Lindlar catalyst in the future, which can be attributed to the fact that this method eliminates the need for the addition of toxic metals. Using 2-Butyne-1,4-diol as an example, this experiment resulted in comparable selectivity between platinum on biomass and platinum on carbon, with percentages of 20%/5%, and conversions of up to 70%. In conclusion, the utilization of Platinum NPs by Escherichia coli presents a novel concept in catalytic alkynol and represents a significant advancement in the field of biochemistry.

### 2.3. Other Noble Metal

**Figure 8:**
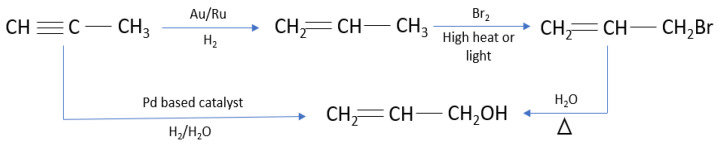
Propargylene-catalyzed synthesis of propenol.

Other noble metals exhibit lower activity compared to Pd in the catalytic hydrogenation of alkynols. Consequently, when utilizing these noble metal catalysts, it is customary to initially hydrogenate the alkynes, to convert them into alkenes (Figure 8). Subsequently, the SN reaction is employed to transform the alkenes into haloalkenes, which are then hydrolyzed to produce alkynols. However, it is evident that this method involves multiple steps and is less productive than employing a Pd catalyst. In this discussion, we will focus solely on the more challenging hydrogenation step in this approach. The theoretical concept behind this catalysis will be elucidated using propylene as an illustrative example.

#### 2.3.1. Au_11_Cl_2_(dppee)_4_]^+^ Nanocluster

The [Au_11_Cl_2_(dppee)_4_]^+^ nanocluster, which exhibits an enhanced symmetry and stability compared to the original Au11 core, enables more efficient semi-hydrogenation. This proposition and its validation using NMR spectroscopy were put forth by Dong et al. [39]. Moreover, they proposed that utilizing this catalyst can yield over 99% conversion and achieve a Z-isomer selectivity above 90% for various alkynes containing ester groups (Table 3).

#### 2.3.2. Ru-Based Catalyst 

Ru catalyst utilizes an alcohol as the hydrogen source and has the ability to generate varying ratios of Z-E isomers, depending on the catalyst type and alcohol employed [40]. This method enables the attainment of yields reaching 99% or higher, while also offering flexibility to cater to the requirements of different isomers. As a result, it provides novel insights and approaches to synthesizing E-form isomers.

## 3. Semi-Hydrogenation of Alkynols over Non-Noble Metal Catalysts

### 3.1. Ni-Based Catalyst

Nickel (Ni), as a representative of non-noble metal catalysts, possesses evident advantages over noble metal catalysts. Employing Ni-based metal catalysts offers a substantial reduction in manufacturing costs and simultaneously ensures high selectivity and conversion rates, establishing Ni as a more promising option than precious metal catalysts in industrial applications. In the following sections, we will examine various aspects of Ni-based catalysts, including Ni-Si catalysts, light-driven catalysis, and the incorporation of additional heteroatoms.

#### 3.1.1. Raney-Ni Catalyst

It is crucial to emphasize that Raney-Ni alone is not a suitable catalyst for alkynol; it is commonly combined with other materials (e.g., Si, N) to efficiently catalyze alkynol. A separate section dedicated to Raney-Ni is justified due to its fundamental role as the foundation for Ni-based catalysts. Murray Raney invented Raney-Ni in 1924 by immersing a 1:1 mixture of Ni-Al alloy in NaOH solution. This process eliminated the aluminum (Al) content, resulting in the formation of porous Ni metal, significantly boosting the catalytic efficiency of Ni for organic compounds. Raney-Ni, known for its affordability, high activity, and applicability as a cathode for hydrogen production, finds extensive utilization in liquid-phase electrocatalysis. Additionally, the leaching process involving Ni_2_Al_3_ after melt quenching and H_2_ pretreatment presents a means to enhance Raney-Ni catalysts and improve their efficiency [41].

#### 3.1.2. Ni-Si Catalyst

**Figure 9:**
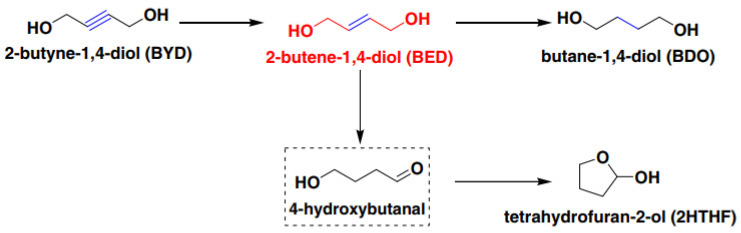
Catalytic hydrogenation of 2-butene-1,4-diol [42]. Copyright 2014, Elsevier.

Ni-Si catalysts employ Si as a heteroatom to modify the semi-hydrogenation reaction. The principle behind this is that, while the addition of Si increases the activation energy of the reaction (thus lowering reactivity), it simultaneously enhances the semi-hydrogenation proportion, resulting in improved selectivity for alkynols. Unlike Lindlar catalysts, this catalyst does not contain any toxic additives, presenting a significant advantage. Experiments conducted by Chen et al. [42] explored the impact of different Si atom additions on selectivity and simultaneously investigated the relationship between contact time and the selectivity of 2-butene-1,4-diol (Figure 9). Determining the optimal contact time with the highest selectivity holds significant importance. Prolonged reaction times lead to the formation of two by-products, namely butane-1,4-diol and tetrahydrofuran-2-ol, resulting in reduced selectivity.

#### 3.1.3. Pristine Ni Catalyst

Ni catalyst used in light-driven semi-hydrogenation reaction is immobilized on a C_3_N_4_ support, employing photocatalysis as the catalytic mode. Importantly, this catalysis utilizes visible light, thus simplifying the reaction conditions significantly and avoiding environmental pollution. The principle behind visible photocatalysis lies in the reduction of the activation energy of semi-hydrogenation by Ni. This occurs through the utilization of visible light energy, which dissociates H ions in solution. Subsequently, the dissociated hydrogen ions partake in an addition reaction with alkynes to synthesize alkynols. Moreover, Jia et al. [43] proposed the exchange of H_2_O with D_2_O using the same mechanism to acquire D-containing enols. This expansion of photocatalysis application extends its scope.

#### 3.1.4. Other Heteroatom Participated Catalysts

Besides utilizing Si as a heteroatom in Ni catalysts, recent studies have explored the incorporation of other heteroatoms such as Al_2_O_3_, N, etc., to achieve selective modulation. The Ni-P catalysts designed by Albani et al. [44] have been thoroughly characterized, and their properties are presented in the Table 4.

### 3.2. Cu- and Zn-Based Catalyst

Among non-noble metal catalysts, Ni exhibits superior catalytic properties. Conversely, non-noble metals like Fe, Co, Cu, and Zn demonstrate lower efficiency as standalone catalysts. Consequently, these metals are commonly employed in bimetallic catalyst formations for enhanced catalytic performance. Here, we will provide brief illustrations using two examples: Cu and Zn.

#### 3.2.1. CuO-Fe_2_O_3_ on SiO_2_ and Al_2_O_3_ Base

As with non-noble metal catalysts, CuO-Fe_2_O_3_ catalysts use inexpensive and readily available metals such as Cu and Fe. This significantly reduces the cost of the catalysts and makes them more amenable to industrial applications [48,49,50].

**Figure 10:**
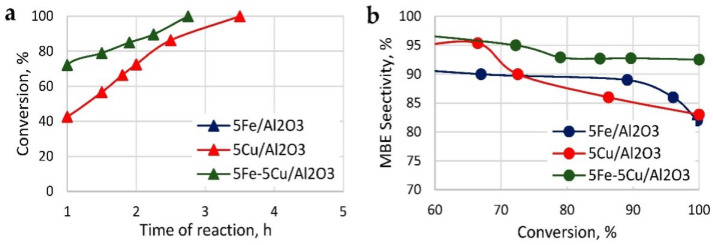
(**a**) Ratio of conversion–time image; (**b**) catalyst used at highest selectivity [51]. Copyright 2021, MDPI.

The mechanism of this reaction is the reduction of 2-methyl-3-butyn-2-ol using nanoparticles formed from iron and copper as the reducing agent for the reaction. In turn, the absorption of hydrogen by 2-methyl-3-butyn-2-ol undergoes a reduction reaction to give 2-methyl-3-buten-2-ol. Iron and copper are oxidized to +2 valent oxides in the reaction, but the presence of copper ions under these conditions causes the +2 valent iron to tend towards the +3 valent form, whereupon the catalyst is transformed to Fe_2_O_3_ and CuO (Figure 10a,b).

Experiments by Anastasiya A. Shesterkina et al. [52] showed that the conversion of 2-methyl-3-butene-2-ol could reach 93% in the presence of γ-Al_2_O_3_ as a supporting substrate (hydrogenation temperature of 150 °C and time of 2.75 h).

#### 3.2.2. Pd-Zn on TiO_2_

Changing the selectivity of the conversion of 2-methyl-3-butyn-2-ol to 2-meth-3-butene-2-ol can be achieved by changing the ratio of Pd to Zn, changing the substrate used, changing the reaction conditions, and altering the reaction conditions (Table 5).

Lyudmila B. Okhlopkova et al. [53] did an experimental comparison of Pd to Zn ratios and different reaction vessels, and they found that 99% selectivity and 96.9% conversion could be achieved with nanoparticles formed by mixing at a 1:1 ratio with 1 as the scaffold substrate.

#### 3.2.3. Cu-Based Catalyst

Despite Cu’s excellent semi-hydrogenation properties, using it as the sole catalyst core reduces its stability. Adding small amounts of other metals to Cu can improve its stability. Zn was chosen by Yuan et al. as an additive to enhance the catalytic effect of Cu. Additionally, a scaffold for this bimetallic catalyst was created using Al_2_O_3_, to ensure its stability in various solutions. The CuZn_3_/HA alloy they produced exhibited 20-times greater stability than the original Cu/HA [52]. Similarly to non-noble metal catalysts, CuO-Fe_2_O_3_ catalysts employ cost-effective and readily available metals, including Cu and Fe, leading to a substantial reduction in catalyst costs and enhancing their suitability for industrial applications [48,49]. The primary mechanism of this reaction involves the reduction of 2-methyl-3-butyn-2-ol using nanoparticles consisting of iron and copper as the reducing agent. Subsequently, the absorbed hydrogen reacts by undergoing reduction, resulting in the formation of 2-methyl-3-buten-2-ol. During the reaction, iron and copper undergo oxidation, forming +2 valent oxides. However, the presence of copper ions in this environment induces the transformation of +2 valent iron towards the more prevalent +3 valent state, subsequently converting the catalyst into Fe_2_O_3_ and CuO.

#### 3.2.4. Zn-Based Catalyst

Bimetals were also utilized by Alberto et al. to enhance catalyst performance. Initially, the metals were combined to form catalysts through colloidal deposition. Subsequently, they evaluated the individual catalytic effects of each combination. Experimental results at 100 degrees Celsius and 1 atm air pressure yielded selectivities of 89% for Pd/ZnO and 94% for Pd-Zn/Al_2_O_3_, respectively. Furthermore, they discovered that a Pt-to-Zn ratio of 3:7 enabled simultaneous high stability and selectivity. This bimetallic catalyst is well-suited for the semi-hydrogenation of alkynels. The incorporation of elemental sulfur in PdZn/Meso-S-C disperses the bimetallic particles, enhances the reaction rate and stability, and marginally improves selectivity and yield over conventional bimetallic catalysts. The experiments conducted by Zhu’s team [54] demonstrated that the catalyst achieved a 97.5% conversion rate of reactants and exhibited 96% selectivity for propargyl alcohol ethoxylates, indicating high catalytic efficiency. Incidentally, they also provided detailed data on the reactions observed during the experiment. However, this method has certain limitations. For instance, the energy-intensive and cumbersome catalyst preparation process, along with the high cost of the PdZn catalyst, pose significant challenges.

**Figure 11:**
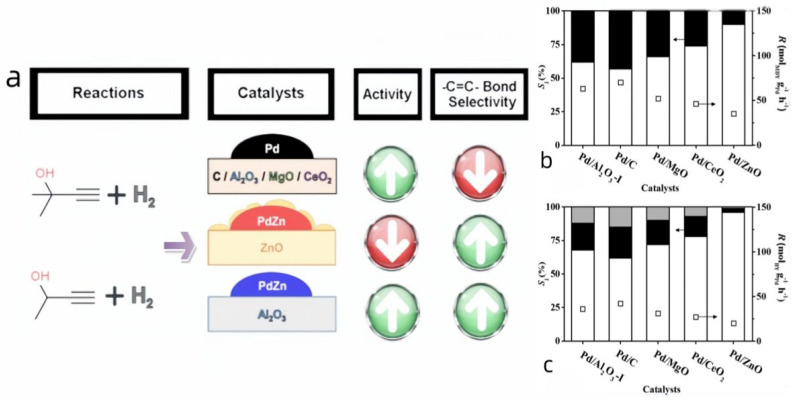
(**a**) Activity and selectivity of different combinations [55]; (**b**) variation in selectivity (Sj, bars) and transformation rate in the hydrogenation of MBY to MBE (open bars) and MBA (solid bars) [55]; (**c**) variation in selectivity (Sj, bars) and transformation rate in the hydrogenation of BY to BE (open bars), BA (solid bars) and BONE (gray bars) [55]. Copyright 2020, American Chemistry Society.

Altering the selectivity of the conversion from 2-methyl-3-butyn-2-ol to 2-meth-3-butene-2-ol can be achieved by adjusting the Pd-to-Zn ratio, varying the substrate, and modifying the reaction conditions. In an experimental comparison conducted by Okhlopkova et al. [53], varying Pd-to-Zn ratios and utilizing different reaction vessels, they discovered that mixing nanoparticles in a 1:1 ratio with 1 as the scaffold substrate yielded a selectivity of 99% and a conversion of 96.9% (Figure 11a–c).

#### 3.2.5. Fe-Based Catalyst

The selection of Fe-based catalysts is also a good choice, which features low cost, low toxicity, and different reactivity. A study by Nikki J. Baka et al. further corroborated the aforementioned features. They simulated a variety of schemes using iron salts. They concluded that the use of iron fluoride salts was the key to obtaining high yields, as FeF_3_-3H_2_O yielded 98% compared to 32% for FeCl_3_ and 26% for Fe(acac)_3_ [56]. We have selected several catalysts with a TOF higher than 5 to be included in the table for industrial production applications (Table 6).

## 4. Comparative Analysis of Metal-Based Catalysts for Alkynol Hydrogenation

To date, numerous catalysts have been developed for the hydrogenation of acetylenic alcohols. Table 7 presents some of these promising catalysts, along with a summary of their data regarding 2-butene-1,4-diol (BED) selectivity and performance metrics/turnover frequency (TOF). The data indicate that Pd-centered catalysts outperform Pt- and Ni-based catalysts. Among these, carbonate supports like Lindlar exhibit notable performance. However, the use of heavy metals can lead to catalyst poisoning, impacting both service life and activity. Pd/C, which serves as an exemplary ligand-enhanced Pd catalyst and a lead (Pb)-free alternative to Lindlar catalysts, exhibits significant selectivity and high activity. This can be primarily attributed to the distinct isolation of its active regions and the electronic nuances stemming from its attached ligands. Research conducted by Nadgeri demonstrated that nanocatalysts can significantly enhance product selectivity and TOF compared to bulk catalysts [27]. Furthermore, Joannet et al. demonstrated that TOF is unaffected by the number of active sites. This finding implies that the influence of liquid–solid mass transport can be disregarded, allowing for the study of intrinsic reaction kinetics [59].

## 5. Conclusions and Outlook

### 5.1. Overview and Contributions

This comprehensive review has explored the selective hydrogenation of alkynols, focusing on both noble and non-noble metal catalysts. The discussion has encompassed a wide range of catalysts, including traditional variants like the Lindlar catalyst and innovative materials like bio-metal composites. By leveraging insights derived from advanced characterization technologies, theoretical principles, and empirical findings, we aimed to unravel the mechanisms that govern selective alkynol hydrogenation.

### 5.2. Discoveries on Noble Metal Catalysts

Pd-based catalysts have demonstrated remarkable efficiency and selectivity in the semi-hydrogenation of alkynols to enols, establishing unprecedented benchmarks. However, optimizing these catalysts requires nuanced modifications, such as the careful addition of stabilizers and inhibitors, which enhance selectivity for intermediates but may slightly reduce catalytic activity. A significant breakthrough is the incorporation of a second metal, such as Zn, In, or Cu. This combination not only affects the geometric and electronic properties of metallic Pd but also finely adjusts substrate and intermediate adsorption, thereby controlling over-hydrogenation. Compared to conventional Lindlar-type catalysts, these Pd-alloys or intermetallic compounds offer a promising avenue, combining high selectivity with the avoidance of toxic additive risks.

### 5.3. Insights on Non-Noble Metal Catalysts

On the other hand, Ni-based materials have emerged as strong contenders, providing a cost-effective pathway for selective hydrogenation of alkynols. The industrial-scale utilization of Raney Ni, albeit under more stringent conditions, highlights its potential. However, the product range in Ni-catalyzed hydrogenation is remarkably influenced by the inclusion of promoters and the inherent acidity of supports. An important challenge, and a potential research avenue, is enhancing the hydrothermal stability of Ni-based catalysts, considering their susceptibility to carbonaceous deposits.

### 5.4. Existing Research Gaps and Challenges

Despite significant progress, certain challenges in the field of alkynol hydrogenation remain unresolved. One crucial aspect is the need for a comprehensive understanding of the structure–property relationships of catalysts. The collaboration between in situ characterization techniques and theoretical computations holds great potential for the future, offering the possibility of resolving the existing uncertainties in the selective catalytic hydrogenation of alkynols.

### 5.5. Future Perspectives and Recommendations

As we look ahead, the importance of maintaining product purity cannot be overstated, particularly due to critical applications in pharmaceutical and polymer precursor synthesis. The quest for effective catalysts, free from toxic residuals, is not simply a desire but a necessity. It is imperative to prioritize the achievement of hydrothermal stability, particularly in aqueous or alcoholic solutions. One intriguing yet under-explored area is the hydrogenation of long-chain alkynols, which plays a pivotal role in pharmaceutical advancements. The upcoming era necessitates a transition towards continuous operations, in response to the growing market demand for enols. As we find ourselves on the verge of these developments, this review serves as both an affirmation of our current knowledge and a resounding call for future endeavors.

## Figures and Tables

**Table 1:** Yield and selectivity data for certain reactions using PdZn/Meso_S-C catalyst **[16]**. Copyright 2022, MDPI.

Number	Reactant	Product	Time (min)	Conv. (%)	Sel. (%)
1	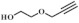	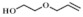	70	97.5	96.0
2	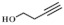	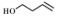	60	92.5	94.7
3	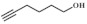	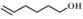	50	>99	95.0
4	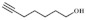	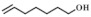	80	>99	94.0
5	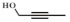	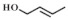	150	93.2	91.7
6	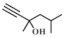	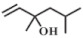	40	85.4	94.7
7	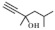	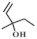	50	97.0	94.4
8	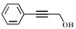	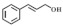	240	83.2	96.9

**Table 2:** Specific properties of each catalysis method.

Specific Properties of Various Thermal Catalysis Methods								
Numbers	Catalyst	Reactants	Temperature (°C)	Pressure (MPa)	Reactor	Alkynol Conversion (%)	Enol Selectivity (%)	Reference
1	Pd-Pb/CaCO_3_	2-Methyl-3-butyn-2-ol	80	0.1	parr autoclave	99	95	[13]
2	Pd/Al_2_O_3_	2-methyl-3-butyn-2-ol	100	0.1	/	99	95	[18]
3	Pd-C	2-methyl-1,4-diol	65	0.3	Büchi stirred	65 (t = 100 min)	73 (t = 100 min)	[12]
4	Pd/ZnO-400	2-methyl-1,4-diol	80	2	parr autoclave	95.8	92.6	[24]
5	PdZn/CN@ZnO	2-methyl-3-butyn-2-ol	35	0.5	stainless steel autoclave	99	96	[25]
6	Pd-Au	2-Methyl-3-butyn-2-ol	30	/	/	98.2	98.9	[26]
7	Pd-C(PVP)	2-Butyne-1,4-diol	100	2.24	/	/	99.6	[27]
8	Pd@PDA@PUF	1-butene 3-Methyl-3-ol	25	0.1	/	98	95	[28]
9	Pd-CaCO_3_	2-Butyne-1,4-dio	50	2.1	parr autoclave	/	99.5	[29]
**Specific Properties of Various Electrocatalysis**								
**Numbers**	**Catalyst**	**Reactants**	**Faradaic** **Efficiency (%)**	**Electric Potential (V)**	**Reactor**	**Alkynol** **Conversion (%)**	**Enol** **Selectivity (%)**	**Reference**
10	Pd-B	2-methyl-3-butyn-2-ol	39.0	−1.0	/	94.5	90.2	[7]
11	Pd	Diphenylacetylene	99(current efficiency)	−0.09	Proton-Exchange Membrane	/	/	[30]
12	Cu	2-methyl-3-butyn-2-ol	95	−0.88	Electrolytic tank	93	97	[20]
13	Pd/carbon nanotubes	Styrene	95	−0.65	oil–water interface	95	/	[31]
**Specific Properties of Photocatalysis**								
**Numbers**	**Catalyst**	**Reactants**	**Temperature (°C)**	**Light Source**	**Reactor**	**Alkynol Conversion (%)**	**Enol** **Selectivity (%)**	**Reference**
14	Pd@Ag/TiO_2_	4-Octyne	/	high pressure mercury lamp	/	99	99	[22]

**Table 3:** Selective semi-hydrogenation of polarized alkynes through stepwise hydride and proton transfers [39]. Copyright 2022, American Chemistry Society. (^a^ Conditions: CH_2_Cl_2_, room temperature, 1 h, [Au_11_HCl(dppee)_4_]^+^. Detailed synthetic procedures are given in the Supporting Information. ^b^ The products were released by adding 1 equiv of diluted HCl (aq.) into the CH_2_Cl_2_ solution of alkenyl-modified gold clusters at room temperature. ^c^ The conversion and selectivity for Z-alkenes were determined by 1H-NMR. Conversion = [alkene]/[alkyne] + [alkene]. Z-selectivity = [Z]/[E] + [Z].)

Number	Reactant	Obtauned ^a^ Cluster	Released ^b^ Product	Conversion ^c^ (%)	Z-Selectivity ^c^ (%)
1	** 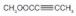 **	** 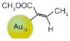 **	** 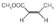 **	>99	91
2	** 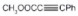 **	** 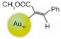 **	** 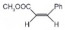 **	>99	90
3	** 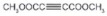 **	** 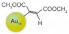 **	** 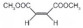 **	>99	99
4	** 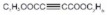 **	** 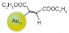 **	** 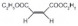 **	>99	97
5	** 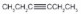 **	None	None	/	/
6	** 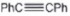 **	None	None	/	/

**Table 4:** Properties of added heteroatom catalysis on Ni.

Numbers	Catalyst	Reactants	Temperature (°C)	Pressure (MPa)	Reactor	Alkynol Conversion (%)	Enol Selectivity (%)	Reference
15	Ni-Si	2-Butyne-1,4-diol	90	3.0	high pressure fixed bed	81.3	82.5	[42]
16	Ni/Al_2_O_3_	2-Butyne-1,4-diol	/	0.1	/	68.9	87.6	[45]
17	Ni-Fe/Al_2_O_3_	2-methyl-1,4-diol	/	0.1	/	82	93	[45]
18	Ni-Ga	ethyne	0	0.1	NMR tubes	67	77	[46]
19	Ni3N	1,2-diphenylethyne	100	2	stainless steel autoclave	99	98	[47]

**Table 5:** Effect of catalyst and reactor type on the activity at 20% conversion and selectivity at 99% conversion in hydrogenation of 2-methyl-3-butyn-2-ol (^a^ reaction conditions: MBY 0.2 M, vg–6.00 mL/min, 1 atm of H_2_, T = 313 K. Coatings were reduced twice at 573 K for 2 h in H_2_ flow (2 mL/min) after evacuation at 573 K under a residual pressure of 13 and after 48 h on stream; ^b^ reaction conditions: MBY/Pd 2100, 50 mL of methanol, 5 atm of H_2_, T = 333 K; ^c^ reaction conditions: MBY/Pd 330, 15 mL of hexane, 1 atm of H_2_, T = 323 K) [53]. Copyright 2018, Elsevier.

Sample	Reactor	A/molMBY/molPd/s	S_99_/%	Y/%
PdZn/TiO_2_	microcapillary	3.0 ^a^	96.9	95.9
PdZn/TiO_2_	batch	1.2 ^b^	74.6	74.5
Pd/TiO_2_	microcapillary	6.1 ^a^	93.9	92.9
Pd/TiO_2_	batch	2.1 ^b^	64.6	65.7
Pd+Pd/CaCO_3_	Batch ^c^	0.09 ^c^	/	94.5

**Table 6:** Some catalysts with TOF higher than 5.

No.	Catalyst	Type of Reaction	TOFs (s^−1^)	Conversion	Reference
1	Raney Ni	Thermos-catalysis	14.7	96.7	[42]
2	Raney 250-NiSi_*x*_		12.2	81.3	
3	Raney 350-NiSi_*x*_		11.6	84.8	
4	Raney 450-NiSi_*x*_		5.2	35.7	
5	Pd_n_/ND@G	Photo-catalytic	10.5	99	[57]
6	Pd/Mg(Al)O-imp	Thermos-catalysis	20.6	90	[58]
7	Pd/Mg(Al)O-nc		12.3	90	

**Table 7:** Metal-based catalysts in terms of their specific activity and selectivity for BED formation during BYD hydrogenation.

Serial No.	Catalyst	Selectivity to BED	TOFs	Reference
1	Raney Ni	67.3	14.7 (s^−1^)	[42]
2	Raney 250-NiSi*_x_*	82.5	12.2 (s^−1^)	
3	Raney 350-NiSi*_x_*	80.4	11.6 (s^−1^)	
4	Raney 450-NiSi*_x_*	89.7	5.2 (s^−1^)	
5	bulk Pd/C catalyst	5	0.29	[27]
6	nanoparticles Pd/C catalyst	99.6	3.1	
7	1% Pd/C	5.0	2.9	[60]
8	1% Pd/Al_2_O_3_	6.0	2.0	
9	1% Pd/MgCO_3_	100.0	0.7	
10	1% Pd/BaCO_3_	100.0	0.7	
11	1% Pd/NH_4_-ZSM-5 10%	100.0	0.6	
12	10% Ni_1% Pd/CaCO_3_	100.0	0.7	
13	1% Pd/CaCO_3_	100.0	1.2	
14	1% Pd/CaCO_3_^a^	83.0	1.5	
15	2 wt% Pd/ACF (active carbon fibers)	97.0	12.0	[59]
16	3 wt% Pd/ACF	97.0	12.0	
17	4 wt% Pd/ACF	97.0	12.0	
18	5 wt% Pd/ACF	97.0	12.0	
19	Pd@PEO-b-P2VP micelles	94.0	0.86	[61]
20	Pd/Mg(Al)O-imp	88.0	20.6	[58]
21	Pd/Mg(Al)O-cop	80	7.6	
22	Pd/Mg(Al)O-nc	86.1	12.3	
23	Pd/Mg(Al)O-ncre	79.4	/	
24	Pd@ZPGly	97.3	79 (h^−1^)	[13]
25	Pd@MonoBor	75.5	466 (h^−1^)	[62]
26	Pd/Al_2_O_3_(Honeycomb)	99.0	71 (h^−1^)	[63]
27	Pd/Al_2_O_3_(Packed-bed)	93.9	125 (h^−1^)	
28	1% Pd/Al_2_O_3_	6.0	2.02 (10^−4^ h^−1^)	[64]
29	1% Pd/C	5.0	2.92 (10^−4^ h^−1^)	
30	5% Pd-2.5% Pd/CaCO_3_	77.0	1.92 (10^−4^ h^−1^)	
31	1% Pt/CaCO_3_^b^	83.0	2.40 (10^−4^ h^−1^)	
32	1% Pt/CaCO_3_	100.0	1.98 (10^−4^ h^−1^)	

^a^ Without ammonia; ^b^ With ammonia.

## Data Availability

Data are contained within the article.
